# Aspiration, but not injection, decreases cultured equine mesenchymal stromal cell viability

**DOI:** 10.1186/s12917-016-0671-2

**Published:** 2016-03-07

**Authors:** Lynn B. Williams, Keith A. Russell, Judith B. Koenig, Thomas G. Koch

**Affiliations:** Department of Clinical Studies, Ontario Veterinary College, University of Guelph, Guelph, Ontario Canada; Department of Biomedical Sciences, Ontario Veterinary College, University of Guelph, Guelph, Ontario Canada

**Keywords:** Equine, Horse, Stem cell, Mesenchymal stromal cell, Umbilical cord blood, Needle, Needle diameter, Stem cell injection

## Abstract

**Background:**

Recently, equine multipotent mesenchymal stromal cells (MSC) have received significant attention as therapy for various conditions due to their proposed regenerative and immune-modulating capacity. MSC are commonly administered to the patient through a hypodermic needle. Currently, little information is available on the effect of such injection has on equine MSC immediate and delayed viability. We hypothesize that viability of equine MSC is not correlated with needle diameter during aspiration and injection.

**Results:**

Using a 3 mL syringe, manual injection of equine cord blood (CB) or bone marrow-derived (BM) MSC with no needle and needles ranging in size from 18 to 30 Ga did not affect immediate MSC viability. Similarly, 24 h post-injection, MSC delayed viability was not different between any of the tested needles as determined by a resazurin-based proliferation assay. Using a 3 mL syringe, aspiration of MSC through 20, 25, and 30 Ga needles resulted in significant decreases in immediate viability with no change in delayed viability when compared to aspiration without a needle. BM- and CB-MSC were observed to be of similar size with a diameter ± SD of 19.8 ± 2.7 and 20.4 ± 2.2 μm, respectively. In comparison, the smallest needles, (30 Ga) have an internal diameter of 160 μm.

**Conclusions:**

Following injection, needle diameter did not affect immediate or delayed viability of equine MSC. Following aspiration through needles sizes 20 Ga and smaller, immediate viability, but not delayed viability, decreased. As a result, an 18 Ga or larger needle should be utilized for aspiration of cell suspensions. In contrast, needle selection for MSC injection should be based on clinical preference and experience rather than concerns over decreasing MSC viability.

## Background

Multipotent mesenchymal stromal cells (MSC)—commonly referred to as mesenchymal stem cells—are thought to have potential as a therapeutic option for orthopedic injuries. In human and veterinary medicine, promising results have been reported *in vivo* following treatment of joint injuries, cartilage defects, and tendon injuries using MSC therapy [1–6]. The current practice of MSC therapy requires administration of a cell-based suspension into the target tissue or structure by injection through a hypodermic needle. In order for stem cells to retain their potential benefits, immediate and delayed viability must not be significantly affected by cell collection, culture, preparation, or injection. The FDA has recommended greater than 70 % viability for cell based therapeutic product prior to injection [7]. Currently there is little reliable evidence as to the effect of aspiration or injection of equine stem cell products through hypodermic needles.

One of the defining characteristics of MSC is their ability to adhere to tissue culture-treated polystyrene plastic. In order for MSC to be used as a therapeutic agent, they must be detached from the culture flask and suspended in liquid prior to injection. This process results in spindle-shaped adherent MSC transforming into spherical free-floating MSC. Reported diameters for suspended rat- and human-derived MSC range from 12–15 μm [8]. The diameter of equine MSC in suspension is not available in the scientific literature. The effect of aspiration and injection on MSC has not been investigated separately. However, intermittent negative pressure was shown to decrease MSC viability in one human wound-healing study [9]. Additionally, Sharp and Mohammad [10] report human red blood cell damage has been reported to increase following injection at pressures greater than those clinically relevant. In this report, injection of whole blood through large diameter needles induced greater red blood cell damage than small diameter needles when injection was performed at a constant pressure [10]. This cellular trauma likely occurs as a result of greater differences in velocity (and hence sheer stress) between fluid in the central (fast flowing) portion of the needle compared to fluid at the slow moving periphery that occur in large diameter needles. Considering pressures well beyond those experienced under clinical conditions were utilized the relevance of this study [10] is unknown. Decreased equine MSC viability has been reported following repeated aspiration and injection of MSC suspensions [11] indicating injection associated trauma can be induced under certain conditions. An increase in apoptotic markers following injection of equine MSC through small diameter needles has been reported to possible sheer stress effects [12]. However, it is undetermined at this point in time if sheer stress affects equine MSC viability since the author’s of the latter study appear to have misinterpreted the above study by Sharp and Mohammad [10]. In the latter study [12] they report that the Sharp and Mohammad study showed increased sheer stress following injection through small diameter needles, when in fact Sharp and Mohammad report increased sheer stress following injection through large diameter needles.

While rat and human bone marrow- (BM-) derived MSC viability did not appear related to needle size or injection rate [8, 13], these findings may not be applicable to equine MSC as numerous species-dependent factors, most notably MSC size, are likely different. This being said, the internal diameter of a 30 Ga hypodermic needle is 160 μm, which is considerably larger than any cell. We therefore hypothesize that viability of suspended equine MSC following aspiration or manual injection is not correlated with needle diameter. The aims of this study were to evaluate MSC immediate and delayed viability 24 h following separate aspiration and injection through a variety of needle sizes commonly used in veterinary medicine.

## Methods

### Preparation of cell suspensions

From nine unrelated horses, 5 BM- and 4 CB-MSC cultures were selected from cryopreserved stock for use in this study. Standard isolation protocols were used in the collection, culture, and cryopreservation of all MSC cultures as described elsewhere [14–16]. BM-MSC cultures had been cryopreserved once and CB-MSC cultures twice prior to commencement of this study. Each cryovial containing 1×10^6^ MSC was thawed in a 37 °C water bath and transferred to either BM-MSC culture media (DMEM low glucose, 10 % fetal bovine serum (FBS), 1 % L-glutamine, and 2 % penicillin/streptomycin) or CB-MSC culture media (containing 30 % FBS, otherwise identical) and cultured at 38 °C, 5 % CO_2_, in a humidified atmosphere to obtain necessary MSC numbers. All MSC cultures were between passage 3–5 and 19–36 days in culture at the time of injection. MSC were detached from culture flasks using trypsin-EDTA and suspended in their respective culture media at a concentration of 5×10^6^ MSC/mL. MSC suspensions were stored in polypropylene vials, at room temperature, in culture media for the duration of the experiment. For injection and aspiration studies MSC cultures were in suspension at room temperature for 12 and 4 h respectively.

### Effect of injection on MSC viability

Using a 3 mL Luer-lock syringe, 0.5 mL of the above cell suspension was injected over a 2 s period into 1.5 mL Eppendorf vials with the needle tip hovering at the top of each vial. One individual (LW) performed injection with the following needles attached to the syringe: no needle, 18Ga x1.5”, 20Ga x1”, 22Ga x1”, 23Ga x3/4”, 25Ga x5/8”, 27Ga x1/2”, and 30Ga x1”. Duplicate injections were performed for each cell population and needle combination. Cell viability was determined using an automated fluorescence-based cell counter (Nucleocounter NC-100, Mandel Scientific Company, Guelph, ON). Each injected cell suspension was seeded into two wells of a 96 well plate at a density of 5000/cm^2^ and cultured for 24 h in 300 μL of respective culture media. Subsequently, a resazurin assay, which had been previously optimized according to the manufacturer’s instructions, was used to assess delayed viability by replacing cell culture media with 300 μL of 10 % resazurin (Sigma-Aldrich, Oakville ON) in phosphate buffered saline. The cells were incubated 4 h before fluorescence was read using an automated plate reader (Spectramax i3, Molecular Devices, Sunnyvale CA) at 585 nm using an excitation wavelength of 555 nm.

### Effect of aspiration on MSC viability

Three BM- and three CB-MSC cultures were selected from the above cell cultures to evaluate the effect of aspiration on MSC viability. MSC suspensions were prepared as described above with the following exception: 0.5 mL of MSC in suspension were slowly aspirated using a 3 mL syringe by one operator (LW) through the following: no needle, 20x1”, 25x5/8”, 30x1” needles, at a rate of 0.25 mL/s. Needles were removed prior to ejection of cell suspensions from the syringe. Immediate and delayed viability were assessed as described above using duplicate samples for each cell suspension/needle combination. Fewer needles were selected for these aspiration studies since no differences were expected based on the injection experiments. In addition, needle size used for aspiration is less critical since the needle used for aspiration is commonly replaced prior to injection.

### Measurement of cell diameter in suspension

The diameter of suspended BM- and CB-MSC was measured by capturing digital images of cell suspension using a digital inverted microscope (EVOS FL, Life Technologies, Carlsbad CA) and measuring cell diameter using software (Image J 1.48, National institutes of health, Bethesda MD, USA) calibrated to the scale bar associated with each image. The diameter of 100 cells was recorded for BM- and CB-MSC cultures with mean and standard deviation (SD) reported.

### Statistical analysis

Viability and fluorescence data was imported into a statistical analysis software package (SAS 9.2, SAS institute, Cary, NC). A general linear model was used to analyze the effect of MSC source (either BM or CB) and needle size using the PROC MIXED function. Residual analysis was performed in order to determine if ANOVA assumptions were met, to detect potential outliers, and evaluate the need for data transformation. Immediate viability (expressed as percentages) for both aspiration and injection portions of the study were analysed following a logit transform with bias correction as follows: logit = log[(r + k)/(n–r + k)] where r viable cells, out of n total cells, and k is the bias correction term, 0.25). Delayed viability for aspiration and injection portions of the study were analysed following a natural log transform.

The residuals were formally tested for normality using the four tests offered by SAS (Shapiro-Wilk, Kolmogorov-Smirnov, Cramer-von Mises, Anderson-Darling) and plotted against the predicted values and variables used in the model. Cell diameters were compared using a two sample t-test (R 3.0.1, R foundation for statistical computing, Vienna, Austria). For the purpose of determining statistical significance, α was set at 0.05.

## Results

### Effect of injection on MSC viability

**Fig. 1:**
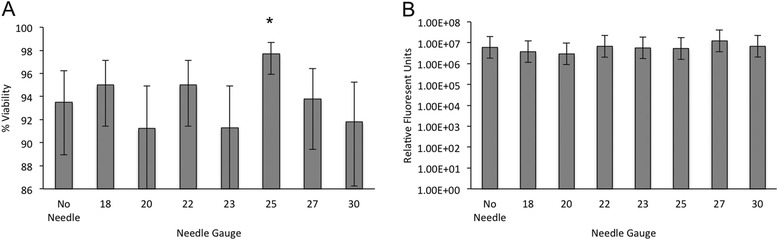
Viability following injection of equine mesenchymal stromal cells; (**a**) Immediate and (**b**) delayed viability of MSC following injection through various needle diameters as assessed using a propidium iodide based automated cell counter and resazurin fluorometric assay, respectively. Differences in viability between MSC tissue sources were not detected, as a result data was combined for analysis. Error bars represent 95 % confidence interval. Asterisks* (*p < 0.05) indicate significant difference from non-injected samples

Regardless of the tissue source or needle size, >89 % immediate viability was achieved. Immediate viability was significantly increased in MSC injected through 25 Ga needles (*p* = 0.01), Fig. 1a. There was no difference between cells originating from BM or CB, and no interaction was detected between MSC source and needle gauge. There were no differences in fluorescence from MSC cultures assessed using the resazurin assay regardless of cell source or needle gauge, and no interaction was detected between MSC source and needle gauge, Fig. 1b.

### Effect of aspiration on MSC viability

**Fig. 2:**
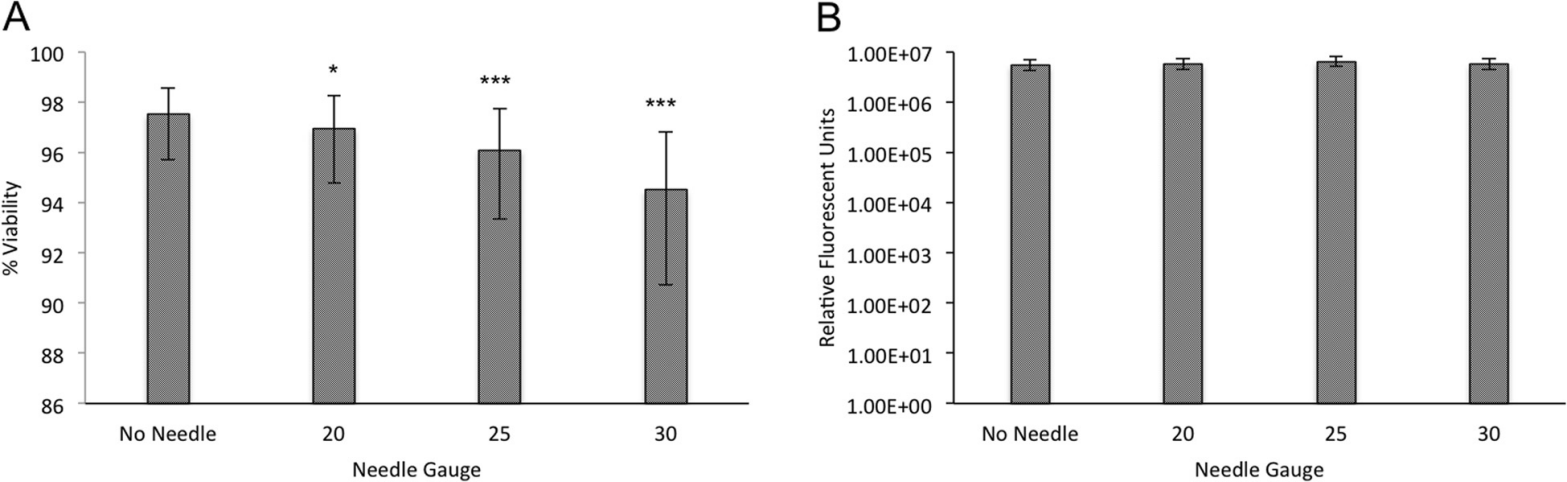
Viability following aspiration of equine mesenchymal stromal cells; (**a**) Immediate and (**b**) delayed viability of equine bone marrow- and cord blood-derived MSC using a propidium iodide based automated cell counter and resazurin fluorometric assay following aspiration through various needle diameters. Differences in viability between MSC tissue sources were not detected, as a result data was combined for analysis. Error bars represent 95 % confidence interval. Asterisks* (*p < 0.05, **p < 0.01, ***p < 0.001) indicate significant difference from non-aspirated samples

Following aspiration, needle size had a significant effect on MSC immediate viability (*p* < 0.0001), but not on delayed viability, Fig. 2. Compared to non-aspirated controls, 20, 25, and 30 Ga needles all decreased MSC viability (*p* = 0.04, 0.0003, and <0.0001, respectively). There was no difference in MSC immediate or delayed viability between MSC from BM and CB origins, and no interaction was detected between MSC source and needle gauge.

### Measurement of cell diameter in suspension

**Fig. 3:**
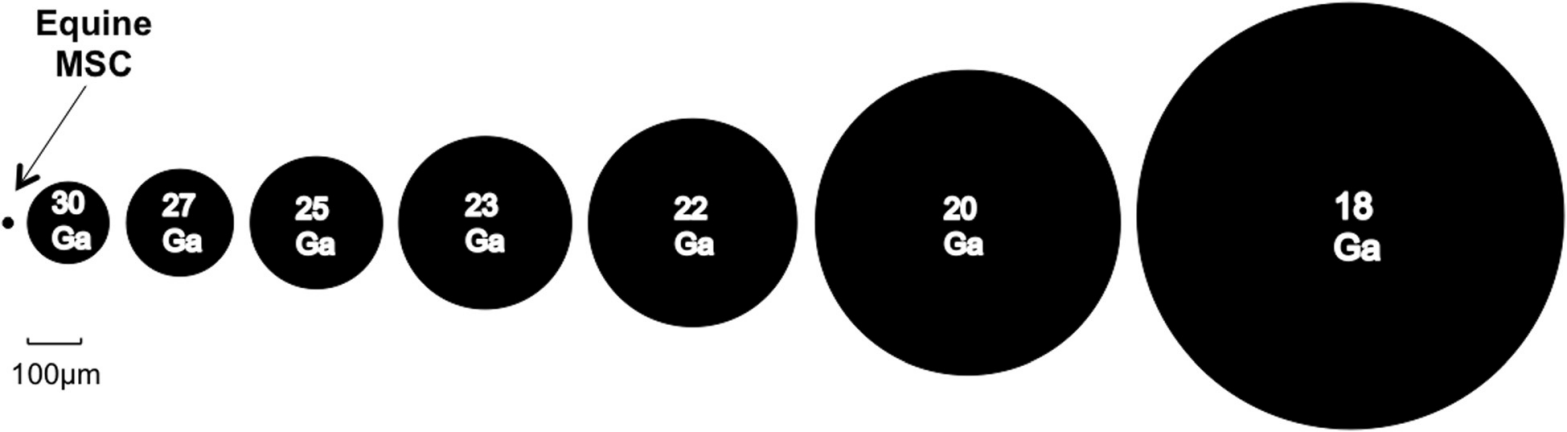
Relative diameter of equine MSC compared to the needles evaluated

No difference was detected between BM- and CB-MSC diameters (*p* = 0.12). Mean ± SD diameters of 19.8 ± 2.7 μm and 20.4 ± 2.2 μm were observed for BM- and CB-MSC respectively, Fig. 3.

## Discussion

Although equine MSC in suspension are >25 % larger in diameter than those of other species [8], needle size did not decrease MSC immediate or delayed viability post-injection. Aspiration, however, significantly decreased MSC immediate viability, but did not affect delayed viability. This finding is of significant interest for clinical administration of equine MSC as care should be taken in selecting a large diameter needle—such as 18 Ga or larger—for aspiration of cell suspensions into the syringe. After the syringe is loaded, however, needle selection can be made without concern for affecting MSC viability.

We observed significantly higher immediate viability in MSC injected through 25 Ga needles compared to all other needles and the no-needle control. The 25 Ga injections were performed sixth in the series of eight injections, which were then repeated for a second technical replicate. As such, we do not consider the experimental setup to have influenced this anomaly. One potential variable that may be responsible for this result is that the 25 Ga needle was one of the shortest needle lengths tested. This may have impacted viability by shortening the duration of any potential cellular injury as MSC were injected through the needle lumen. Differences in needle lengths may have affected our results by varying the duration of injection or aspiration forces acted on MSC. The needles evaluated however, are commonly available in veterinary medicine and as such more likely to be used in everyday use. Considering there are no clinical implications for increased MSC viability and that this increase was noticed using the third-smallest diameter needle evaluated, the increased viability in this group is not considered clinically significant.

The injection rate used in this study is likely slower than the rates used in clinical practice where larger gauge needles (18–22 Ga) are used. These rates were, however, the maximum that could be achieved using small diameter needles (<25 Ga). Significant manual force was required to achieve the desired injection rate with a 25 Ga and 27 Ga needle and the rate of 0.25 mL/s could not be achieved using a 30 Ga needle despite maximal force (to the extent of bending the syringe plunger). The use of progressively smaller needles compensated for the relatively slow injection rate used and allows us to conclude that the forces achieved by MSC injection using a 3 mL syringe are not sufficient to decrease MSC viability or proliferative ability. As smaller syringes were not evaluated, no inference can be made about any potential damage that injection through these syringes may induce on equine MSC. It is possible that a syringe with a smaller plunger may be able to create sufficient pressure and shear forces on MSC during injection to decrease immediate or delayed MSC viability. Garvican et al. [12] recently reported equine BM-MSC injected using a 2 mL syringe show increased propidium iodide and annexin V staining (necrosis and apoptosis, respectively) immediately following injection through 21 or 23 Ga needles. Differences in methodology are likely responsible for the differences observed as higher pressures, longer needles, increased injection rates, and cellular trauma associated with negative pressure (aspiration through the test needle) [9] may have influenced their results. Conflicting results have been reported as to the effect of injection through various needle diameters using human and rat MSC. The most dramatic decreases in MSC viability, increases in markers of apoptosis, and decreased MSC proliferation were observed using MSC concentrations as high as 5x10^7^/mL, rapid injection rates, small diameter needles and storage for more than 2 h at room temperature prior to injection [17].

In previous studies aspiration through the test needle prior to injection [12, 17, 18] or repeated aspiration and injection [11] likely also have a negative effect on MSC viability due to intermittent negative pressure which has been associated with apoptosis in other studies [9]. Consistent with the observation of detrimental effects on MSC viability in other species, immediate injection without aspiration of dilute cell suspensions at slow rates resulted in no change in MSC viability or apoptotic fraction using needles as small as 30 Ga [8, 13]. While performing the injection studies, we observed that pre-injection MSC viability had decreased by approximately 10 % towards the end of data collection. We attributed this to the time that MSC suspensions had been kept at room temperature (~12 h) and, therefore, elected to decrease the number of needles and cell suspensions evaluated for subsequent aspiration studies to reduce the time MSC were at room temperature. We considered this a reasonable strategy since in a clinical situation the needle used to aspirate a cell suspension is routinely changed prior to injection. We conclude that evaluation of 18 Ga needles should have been included in the aspiration study due to the significant decrease in viability in all needles tested. However, although statistical significance was detected between MSC aspirated through 20 Ga needles (*p* = 0.04) and MSC aspirated without a needle, immediate viability only decreased by 0.6 % on average from 97.6 to 97.0 %. This is most likely not of clinical significance, and viability remained well above the 70 % cut-off suggested by FDA.

Our methodology assessed immediate viability by propidium iodide exclusion by the intact cellular membrane and delayed viability through metabolism of the dye resazurin. Both of these measurements provide information as to the viability and physiological state of the undifferentiated MSC, but do not assess differential potency or immunomodulatory properties of the MSC. We do not expect these functional properties of the MSC to be affected, but future work could determine if aspiration or injection affects the ability of the MSC to differentiate into adipogenic, osteogenic or chondrogenic cell fates or their lymphocyte suppressive properties in vitro.

This study is limited in that the methods employed only evaluated membrane integrity and gross metabolic activity. More sensitive methods of evaluating individual cell metabolic activity or other measures of cellular health, such as markers of apoptosis, may have detected additional differences. Although the needles evaluated were lengths commonly available in veterinary medicine different gauge needles were not all uniform in length. As such the duration of any adverse forces varied between the various test needles. This could have been avoided by utilizing needles of a standardized length, possibly at the expense of clinical utility.

## Conclusion

Care should be taken to use large diameter needles when aspirating equine MSC. However, for injection, needle selection should be based on clinical experience, knowledge of patient behavior, and the anatomical relationship of the targeted structure without concern over MSC viability.

### Ethics approval

This study was specifically approved by the University of Guelph Animal Care Committee with regard to the collection of equine CB (animal use protocol 1570). Additional research conducted using specimens of this kind does not require review by the Animal Care Committee (falls under CCAC Category of Invasiveness A) and therefore this study can be considered to have been conducted in accordance with institutional ethics guidelines. No animals were sacrificed during the study. Equine umbilical cord blood was collected on two privately owned commercial farms in Southern Ontario. Four of five samples were collected on one farm from Thoroughbred foals. One sample was collected on another farm from a Warmblood foal. Informed consent was obtained in writing from the horse owners/agents prior to sampling. The broodmares on the foaling farms are housed in large foaling boxes. Both farms are staffed 24/7 and mares are under constant video surveillance and carrying foaling alarms to allow for observed foaling and assisted delivery if needed. Umbilical cord blood was collected by the farm staff after receiving instruction by Dr. Koch. Instruction included video-review of cord blood collection. Cord blood was collected from an isolated segment of the umbilical cord after the umbilical cord had been clamped and detached from the foal. Three of five BM samples were collected immediately following euthanasia from animals euthanized for reasons unrelated to this study. The remaining two BM-MSC cell cultures were obtained from excess cultured BM-MSC of client owned horses. In this instance, animal owners signed consent forms acknowledging BM-MSC in excess to the numbers agreed upon would be donated for research purposes. Since the methods used for acquisition of BM samples did not require review by the Animal Care Committee (falls under CCAC Category of Invasiveness A), this study has been conducted in accordance with institutional ethical guidelines.

### Consent for publication

Not applicable.

### Availability of data and material

Data for this study has not been uploaded to a repository but are available upon request.

## Abbreviations

ANOVA: analysis of variance
BM: bone marrow
CB: cord blood
FDA: Food and Drug Administration
MSC: mesenchymal stromal cell
